# Eating-Related Profiles in Multiple Sclerosis: Associations with Coping, Health Locus of Control, and Self-Management

**DOI:** 10.3390/nu18182984

**Published:** 2026-09-11

**Authors:** Maciej Wilski, Jarosław Gabryelski, Waldemar Brola

**Affiliations:** 1 Department of Health and Rehabilitation Psychology, Poznań University of Physical Education, 61-871 Poznan, Poland; 2 Division of Universal Design and Mobility Devices, Institute of Transport, Poznan University of Technology, 61-138 Poznan, Poland; jaroslaw.gabryelski@put.poznan.pl; 3 Collegium Medicum, Jan Kochanowski University, 25-317 Kielce, Poland; wbrola@wp.pl

**Keywords:** multiple sclerosis, eating-related tendencies, emotional eating, dietary restraint, latent class analysis, coping, health locus of control, self-management

## Abstract

**Background/Objectives:** Eating-related difficulties in people with multiple sclerosis (MS) may reflect different behavioural and psychological processes, including emotional responses to illness-related stress, habitual overeating, attempts to control food intake, and broader self-management routines. Variable-centred analyses may obscure this heterogeneity by examining eating-related tendencies separately. This study aimed to examine whether habitual overeating, emotional overeating, and dietary restraint in adults with MS are better represented as one general continuum of eating-related difficulty or as distinct person-centred profiles that differ in coping styles, health locus of control, and MS self-management. **Methods:** This cross-sectional secondary analysis included 382 adults with definite MS. Latent class analysis used 30 binary My Eating Habits Questionnaire items. One-step latent class regression models examined coping and health locus of control. Linear regression compared self-management scores. The models were adjusted for sex, age, disease duration, and Expanded Disability Status Scale score. **Results:** A five-class solution was retained (BIC = 11,067.60; entropy = 0.920), comprising low difficulties, moderate mixed tendencies, two emotional eating/restraint profiles, and dysregulated overeating. Emotional eating with moderate restraint was associated with lower task-oriented coping and internal health control, and with higher emotion-oriented and avoidance-oriented coping and external health-control beliefs. Dysregulated overeating was associated with lower task-oriented coping, higher avoidance-oriented coping, and stronger powerful-others health beliefs. Self-management was higher for emotional eating with moderate restraint (B = 5.3, 95% CI 1.8 to 8.9; *p* = 0.003) and high emotional eating and restraint (B = 6.0, 95% CI 1.8 to 10.3; *p* = 0.005), versus low difficulties. Sensitivity analyses gave similar findings. **Conclusions:** Eating-related tendencies in MS formed distinct profiles rather than a single continuum. These profiles differed in coping and health-control patterns, suggesting that similar eating-related difficulties may occur in different psychological and self-management contexts. The higher self-management scores observed in restraint-related profiles should be interpreted cautiously and should not be taken to indicate that restrictive eating is adaptive. This study does not establish clinical classification or treatment recommendations. Instead, it provides a descriptive empirical basis for future studies testing whether these profiles are stable and whether they predict dietary, nutritional, psychological, and clinical outcomes.

## 1. Introduction

Multiple sclerosis (MS) is a long-term neurological condition in which everyday disease management extends beyond pharmacological treatment to a broad set of self-management behaviours, including symptom monitoring, maintaining health, using disease-related information, communicating with healthcare professionals, engaging in rehabilitation-related activities, and adapting to changing functional demands [1,2]. Within this broader context, dietary and eating-related behaviours may be considered part of lifestyle self-management in MS rather than merely an aspect of diet, particularly because nutritional behaviours in MS have been linked with self-efficacy, physical activity, and communication with physicians [3]. However, eating-related tendencies in chronic illness may have different behavioural meanings. For some individuals, they may reflect emotional responses to stress or functional limitations; for others, they may be linked to routines, attempts to control body weight, or broader patterns of monitoring and self-regulation. This creates an important descriptive problem for MS research: similar levels of eating-related difficulty may reflect different combinations of behavioural tendencies rather than a single underlying severity dimension. The present study does not address eating-disorder diagnoses. Instead, it focuses on eating-related tendencies that may occur in the general population and may be especially meaningful when long-term illness creates recurrent emotional, functional, and self-regulatory demands.

The My Eating Habits Questionnaire distinguishes three such tendencies: habitual overeating, emotional overeating, and dietary restraint [4]. The MEH was selected for this secondary analysis for both substantive and methodological reasons. Substantively, its three domains corresponded to the eating-related tendencies central to the present research question: repeated overeating in everyday situations, eating in response to emotions, and intentional restriction of food intake. Methodologically, the MEH consists of 30 dichotomous items, which allowed item-level response configurations to be modelled directly using Bernoulli latent class analysis rather than relying on subscale totals. Emotional eating refers broadly to changes in food intake in response to affective states, although its expression may depend on the type and intensity of emotion, individual appetitive regulation, and contextual factors [5]. Prospective and cross-sectional evidence also links stronger emotional eating with adverse weight-related outcomes, particularly in the presence of depressive symptoms and other vulnerability factors [6]. Dietary restraint, in contrast, reflects intentional attempts to limit intake or manage body weight. It may coexist with overeating tendencies rather than simply representing their opposite. Accordingly, an individual who reports substantial restraint may also report emotionally triggered or habitual overeating, and the clinical meaning of either tendency may depend on the broader pattern in which it occurs.

Most research on eating-related tendencies remains variable-centred: dimensions are analysed separately, and associations are interpreted one scale at a time. This approach can overlook meaningful heterogeneity in the way eating-related tendencies co-occur within individuals. Person-centred methods, including latent class analysis (LCA), instead identify subgroups defined by shared configurations of item responses while preserving the probabilistic nature of class membership [7]. Such an approach is particularly suitable when the indicators are dichotomous questionnaire items, as in the My Eating Habits Questionnaire. It allows the analysis to move beyond the question of whether an average score is higher or lower, and toward the more precise descriptive question of whether qualitatively distinct patterns of eating-related behaviour can be identified.

Studies in children and adults support the value of this perspective. Person-centred analyses have identified eating profiles characterised by comparatively high food approach, high food avoidance, broadly moderate eating, or more selective combinations of appetitive traits; these profiles have differed in weight status, temperament, psychological distress, and food-related quality of life [8,9]. Research focused on emotional, disinhibited, and restrained eating has likewise identified profiles that differ in depressive symptoms, impulsivity, body esteem, and other psychological characteristics [10,11]. These studies do not imply that profile structures will be identical in MS. Rather, they illustrate that behaviours conventionally represented as separate dimensions may form configurations with different psychological correlates that should be examined prospectively in disease-specific cohorts.

Coping styles and health locus of control are particularly relevant variables for characterising eating-related profiles in MS. Coping reflects the cognitive and behavioural efforts used in response to stress, whereas health locus of control concerns whether people attribute health outcomes primarily to their own actions, powerful others, or chance. Both constructs have been associated with psychological adjustment in MS, and their relationships with mental health and self-management are not fully explained by neurological disability alone [12,13]. In parallel, MS self-management is a clinically meaningful outcome because it incorporates health-maintenance behaviour, disease-related knowledge and information seeking, communication with healthcare providers, and treatment-related behaviours and barriers [2]. The relation between eating-related tendencies and self-management is not necessarily unidirectional. For example, dietary restraint may accompany deliberate health-maintenance behaviours in some individuals but may coexist with distress-related eating in others. Therefore, self-management was considered an important, but empirically open, characteristic of the identified classes.

From the perspective of MS care, an unresolved question is whether self-reported overeating, emotional eating, and dietary restraint in people with MS should be understood as indicators of one general level of eating-related difficulty, or as distinct combinations of tendencies that occur in different coping and health-control contexts. Without such information, clinicians and researchers lack an empirical basis for deciding whether future assessment and intervention studies should treat eating-related difficulties in MS as a single continuum or as separable behavioural profiles. The present analysis begins to address this question descriptively by identifying item-level profiles of eating-related tendencies and examining whether these profiles differ in coping, health locus of control, and self-management.

To our knowledge, no previous study has used item-level LCA to examine configurations of habitual overeating, emotional overeating, and dietary restraint among people with MS. The present study, therefore, had three aims. First, we used all 30 dichotomous items of the My Eating Habits Questionnaire to identify latent classes of eating-related tendencies in adults with MS. Second, we examined adjusted associations of class membership with task-oriented, emotion-oriented, and avoidance-oriented coping, as well as internal, powerful-others, and chance health locus of control. Third, we examined whether MS self-management differed across the identified classes. We expected classes marked by elevated emotional eating and/or dysregulated overeating to show greater emotion-oriented and avoidance-oriented coping, lower internal health control, and stronger external health-control beliefs than a low-difficulties class. Because the implications of combined restraint and overeating tendencies for self-management were less clear, analyses of self-management were regarded as exploratory. Given the cross-sectional design, all observed relationships were interpreted as associations rather than evidence of temporal or causal effects.

### Participants and Procedure

This cross-sectional secondary analysis used data from a broader questionnaire study of psychosocial functioning, self-management, and health-related outcomes among people with multiple sclerosis (MS). The parent study was conducted in cooperation with two specialist clinical centres in Poland: the Multiple Sclerosis Rehabilitation Centre in Borne Sulinowo and the Department of Neurology at the Specialist Hospital in Końskie. The participants were recruited between December 2015 and January 2017. The dataset and core study procedures have been reported in previous publications from this cohort [12,13].

The analytic sample included 382 adults with definite MS (256 women and 126 men). The participants were recruited during routine control visits. The eligibility criteria were a neurologist-confirmed diagnosis of definite MS, according to the revised McDonald criteria [14], no clinical relapse or MS exacerbation during the preceding 30 days, and the ability to provide written informed consent and complete the self-report questionnaire independently. Individuals with a documented psychotic disorder or clinically apparent cognitive impairment were not invited when the treating neurologist or research staff considered that these conditions could compromise informed consent or reliable questionnaire completion. Co-occurring somatic conditions were not blanket exclusion criteria unless they were judged likely to preclude questionnaire completion or substantially affect participation. Of the 400 patients initially selected for participation, 18 were excluded from the analytic dataset because of incomplete questionnaire data, leaving an analytic sample of 382 participants. Because the present latent class analysis required complete data on the MEH indicators and external correlates, these participants were excluded using complete-case analysis. Detailed comparisons between the excluded and included participants were not possible because complete demographic and clinical data were not retained for all of the excluded cases.

Eligible patients received verbal information about this study from a member of the research team. Those who agreed to participate provided written informed consent and were assured that their data would remain confidential. The participants completed the questionnaire battery individually in a quiet room during a routine outpatient clinic visit. The questionnaire forms were checked for completeness immediately after return, and the participants were invited to complete any unintended omissions whenever possible. Clinical information, including MS subtype and neurological disability measured using the Expanded Disability Status Scale (EDSS), was recorded by a neurologist.

The parent study was conducted in accordance with the Declaration of Helsinki. The Bioethics Committee at Poznan University of Medical Sciences confirmed that formal ethical approval was not required for this non-interventional questionnaire-based study. The present work is a secondary analysis of the complete-case dataset and focuses on latent classes of eating-related behaviours and their associations with coping, health locus of control, and self-management.

## 2. Measures

### 2.1. Eating-Related Tendencies

Eating-related tendencies were assessed using the My Eating Habits Questionnaire (MEH; Moje Zwyczaje Żywieniowe) developed by Ogińska-Bulik and Putyński [4]. The MEH is a Polish self-report questionnaire comprising 30 dichotomous yes/no items. It includes three 10-item dimensions: habitual overeating, emotional overeating, and restrictive dieting. In accordance with the scoring key, a response indicating the relevant eating-related tendency was coded as 1 and the alternative response as 0. For five reverse-keyed items (items 2, 5, 13, 17, and 23), a “no” response was coded as 1; for all remaining items, a “yes” response was coded as 1. Higher subscale sums indicated greater endorsement of the corresponding tendency. The dichotomous item format was retained because the aim of the present analysis was to identify item-level patterns of endorsed eating-related tendencies rather than to compare mean subscale scores.

All 30 binary item responses were entered as indicators in the latent class analysis. Thus, class membership was derived from response configurations across individual items rather than from pre-defined subscale totals. The original three-domain structure was used to aid substantive interpretation and labelling of the identified classes. Internal consistency in the present sample was acceptable for habitual overeating (Cronbach’s α = 0.81) and emotional overeating (α = 0.80), and lower but acceptable for restrictive dieting (α = 0.68). The MEH is not a diagnostic instrument for eating disorders; it was used to characterise eating-related behavioural tendencies.

### 2.2. Coping Styles

Coping styles were assessed using the Coping Inventory for Stressful Situations (CISS) developed by Endler and Parker [15]. This 48-item questionnaire asks respondents to indicate how frequently they engage in different coping responses when confronted with stressful situations. Items are rated on a five-point Likert-type scale. The CISS yields three 16-item scores: task-oriented coping, emotion-oriented coping, and avoidance-oriented coping. Each score ranges from 16 to 80, with higher scores indicating greater use of the respective coping style. In the present sample, Cronbach’s α values were 0.79 for task-oriented coping, 0.88 for emotion-oriented coping, and 0.69 for avoidance-oriented coping.

### 2.3. Health Locus of Control

Health locus of control was measured using the 18-item Multidimensional Health Locus of Control (MHLC) Scale developed by Wallston and colleagues [16]. The instrument comprises three six-item dimensions: internal health locus of control, powerful-others health locus of control, and chance health locus of control. The participants rate agreement with each statement on a six-point Likert-type scale, and scores on each dimension range from 6 to 36; higher scores reflect stronger endorsement of the respective health-control belief. Internal consistency coefficients in the present sample were α = 0.64 for internal control, α = 0.81 for powerful-others control, and α = 0.63 for chance control.

### 2.4. Multiple Sclerosis Self-Management

Multiple sclerosis self-management was assessed using the 24-item Multiple Sclerosis Self-Management Scale-Revised (MSSM-R) developed by Bishop and Frain [2]. Items are scored on a five-point response scale ranging from 1 (disagree completely) to 5 (agree completely), yielding a total score from 24 to 120; higher scores indicate greater self-management. The total score showed good internal consistency in the present sample (Cronbach’s α = 0.89).

The validated Polish version of the MSSM-R [17] recommends the use of the total score rather than interpretation of the original subscales because the original factor structure was not reproduced in the Polish validation study. Therefore, the full 24-item total score was used in the primary analysis. Because one MSSM-R item refers to maintaining regular meals and could conceptually overlap with the eating-related indicators, a prespecified sensitivity analysis repeated the class comparisons after excluding that item. This 23-item score was used only for sensitivity analysis and was not treated as a separate validated scale.

### 2.5. Sociodemographic and Clinical Characteristics

Sex, age, and disease duration were obtained from the standardised questionnaire used in the parent study. Disease duration was expressed as years since MS diagnosis. MS subtype and neurological disability were determined by a neurologist. Neurological disability was assessed using the Expanded Disability Status Scale (EDSS) [18], which ranges from 0 (normal neurological examination) to 10 (death due to MS), with higher scores reflecting greater disability. Sex, age, disease duration, and EDSS score were included as covariates in the adjusted analyses. The MS subtype and other available clinical characteristics were used to describe the latent classes.

### 2.6. Statistical Analysis

The analytic sample comprised 382 participants. The latent class analyses and one-step latent class regression models were estimated using Mplus, version 8.10 (Muthén & Muthén, Los Angeles, CA, USA). The MEH items were specified as categorical indicators and modelled as binary indicators of latent class membership. The models were estimated using maximum likelihood estimation for the categorical latent class models. To reduce the risk of convergence to local maxima, each model was estimated using multiple random sets of starting values. For the retained five-class solution, the best log-likelihood was replicated in 55 of 250 random starts. Model convergence was assessed using the standard Mplus convergence criteria, and solutions were evaluated for inadmissible parameter estimates and substantive interpretability. The remaining analyses, including descriptive statistics, unadjusted class comparisons, self-management regression models, and pseudo-class sensitivity analyses, were conducted using IBM SPSS Statistics for Windows, version 29.0 (IBM Corp., Armonk, NY, USA). All of the analyses were conducted using complete cases. No imputation was performed because the analytic sample included only participants with complete data on the MEH items, external correlates, self-management variables, and covariates included in the present analyses. Continuous variables were summarised as means and standard deviations (SDs), and categorical variables as frequencies and percentages. Unadjusted differences in demographic and clinical characteristics across eating-related classes were examined using one-way analysis of variance for continuous variables, Pearson chi-square tests for categorical variables with adequate expected cell counts, and a Monte Carlo exact test for MS clinical course. All of the tests were two-sided, and *p* < 0.05 was considered statistically significant.

Latent class analysis (LCA) was conducted using the 30 dichotomous items of the My Eating Habits Questionnaire (MEH) as class indicators [7]. Bernoulli LCA models with one through seven classes were estimated. The five-class solution was retained because it had the lowest Bayesian information criterion (BIC), high classification quality, no trivial class, and a substantively coherent pattern of eating-related tendencies. Model selection also considered the Akaike information criterion (AIC), sample-size-adjusted BIC (SABIC), entropy, estimated class sizes, replication of the best log-likelihood, and the interpretability of conditional item-response probabilities. Because information criteria may not converge on the same solution, the final model was selected using a combined statistical and substantive approach, with priority given to BIC, adequate class size, classification quality, and substantive interpretability. For descriptive analyses and the primary self-management models, the participants were assigned to their modal class using the highest posterior membership probability.

Associations of coping styles and health locus of control with latent-class membership were examined in two separate one-step latent class regression models. The first model included task-oriented, emotion-oriented, and avoidance-oriented coping scores from the Coping Inventory for Stressful Situations (CISS); the second included internal control, powerful-others, and chance dimensions from the Multidimensional Health Locus of Control scale (MHLC). Both models were adjusted for sex, age, disease duration, and Expanded Disability Status Scale (EDSS) score. The CISS and MHLC scores were standardised before modelling; therefore, relative risk ratios (RRRs) with 95% confidence intervals (CIs) represented associations with membership in each class per 1-SD higher predictor score. The low eating-related difficulties class served as the reference category. Global associations were evaluated using 4-df Wald tests.

Analyses of external correlates were exploratory and were intended to characterize the latent classes rather than to test a single confirmatory hypothesis. Therefore, no formal correction for multiple testing was applied. Interpretation focused on global tests, effect estimates, confidence intervals, and the consistency of patterns across related variables rather than on isolated class-specific *p*-values.

Associations between eating-related class membership and multiple sclerosis self-management were estimated using multivariable linear regression models adjusted for sex, age, disease duration, and EDSS. The primary outcome was the validated 24-item total score of the Multiple Sclerosis Self-Management Scale-Revised (MSSM-R). Adjusted means were estimated at the sample mean values of the covariates, and class-specific contrasts were expressed as unstandardized regression coefficients with 95% CIs, using the low eating-related difficulties class as the reference group. Incremental R^2^ was calculated as the increase in explained variance after adding the 4-df class term to the covariate-only model.

Classification uncertainty in the self-management comparisons was examined in a pseudo-class sensitivity analysis. Across 2000 draws, class assignments were sampled from each participant’s posterior probability vector, the covariate-adjusted linear regression was re-estimated, and estimates and variance components were pooled using Rubin’s rules [19].

Because MSSM-R Item 7 assesses regular meal consumption and could overlap conceptually with the eating-related class indicators, the self-management analyses were repeated after excluding this item from the total score. Given the cross-sectional design, all findings are interpreted as associations and not as evidence of temporal or causal effects.

## 3. Results

### 3.1. Latent Class Solution and Class Characteristics

The fit indices for the one- through seven-class solutions are presented in Table 1.

The five-class solution was retained because it had the lowest Bayesian information criterion (BIC = 11,067.60), high classification quality (entropy = 0.920), and an adequate smallest estimated class (n = 31.0; 8.1% of the sample). The BIC values were higher for the four-class (11,093.94), six-class (11,112.64), and seven-class (11,155.60) solutions. Although the six- and seven-class models improved the log-likelihood, AIC, and SABIC, they did not improve the BIC and mainly subdivided existing response patterns without yielding clearly distinct or behaviourally more interpretable profiles. They also produced smaller classes that were less suitable for subsequent analyses of external correlates. Therefore, the five-class model was preferred as the most parsimonious solution supported by BIC, classification quality, adequate class size, and substantive interpretability.

Classification quality for the retained five-class model was high. The mean maximum posterior membership probability was 0.951, and only 5.2% of the participants had a maximum posterior probability below 0.70. Class-specific average posterior probabilities for modal class assignment were 0.926 for low eating-related difficulties, 0.946 for moderate mixed tendencies, 0.975 for emotional eating with moderate restraint, 0.934 for high emotional eating with restraint, and 0.997 for dysregulated overeating. These values indicate acceptable-to-excellent classification precision, including for the smallest dysregulated overeating class.

**Figure 1:**
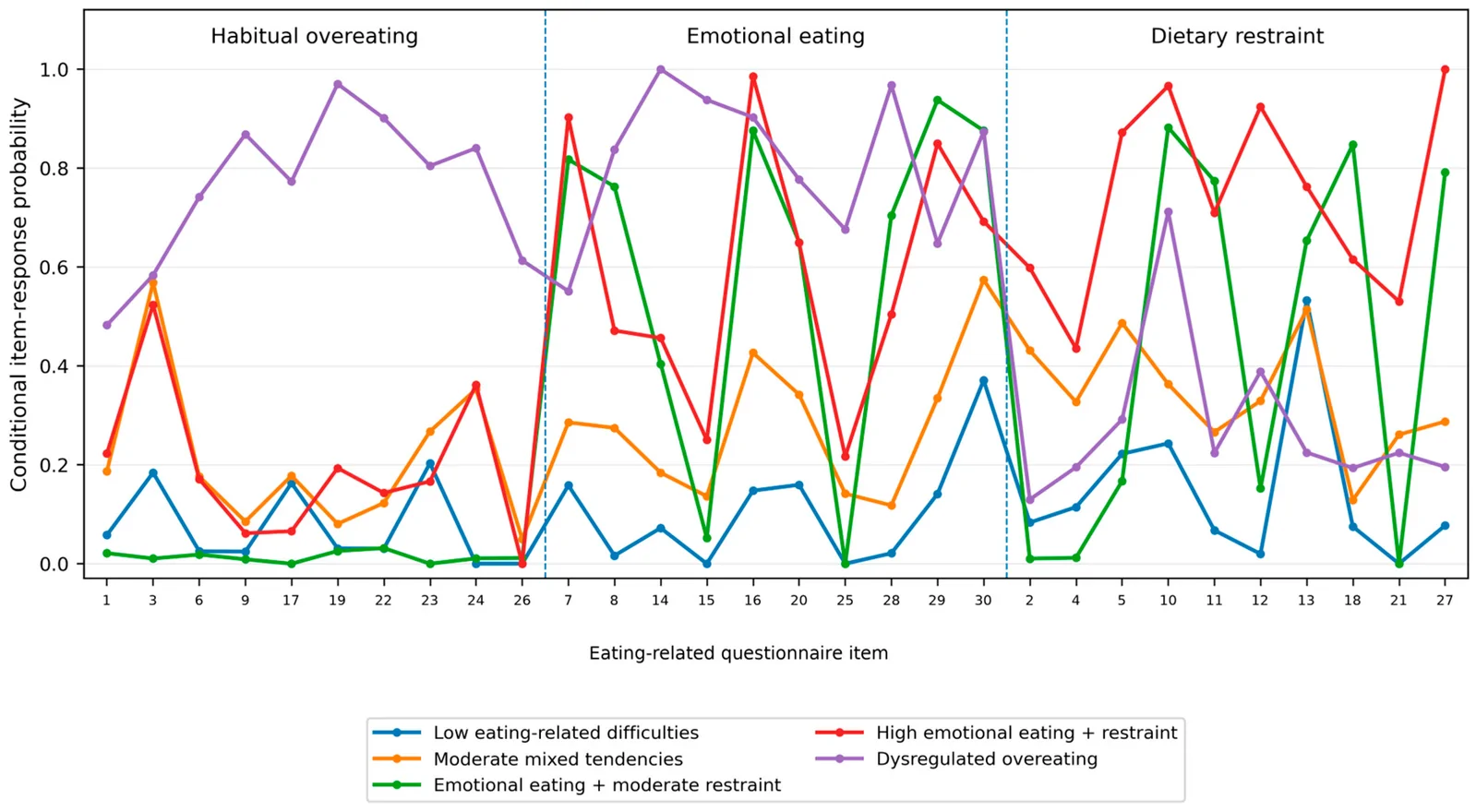
The conditional item-response probabilities for the five-class solution (N = 382). The lines represent the probability of endorsing each dichotomous eating-related questionnaire item within each latent class. The questionnaire items, identified by item number, are grouped by habitual overeating, emotional eating, and dietary restraint. The class labels were assigned on the basis of the characteristic patterns of item-response probabilities.

Conditional item-response probabilities for the retained solution are shown in Figure 1. The first class, labelled as low eating-related difficulties, showed consistently low probabilities of endorsing items across habitual overeating, emotional eating, and dietary restraint. The second class, moderate mixed tendencies, was characterised by intermediate endorsement probabilities across all three domains. The third class, emotional eating with moderate restraint, showed high probabilities of emotional-eating items, low probabilities of habitual-overeating items, and moderate endorsement of dietary-restraint items. The fourth class, high emotional eating and restraint, showed high probabilities of endorsement across emotional-eating and dietary-restraint items. The fifth class, dysregulated overeating, was characterised by high probabilities of both habitual-overeating and emotional-eating items, with comparatively lower endorsement of dietary restraint. These class labels are descriptive summaries of item-response patterns and should not be interpreted as diagnostic categories or eating-disorder classifications.

Table 2 presents the unadjusted demographic and clinical characteristics according to modal class membership. The classes differed in sex distribution (*p* = 0.022), disease duration (*p* = 0.002), EDSS score (*p* < 0.001), and MS clinical course (Monte Carlo *p* = 0.006), whereas the mean age did not differ across classes (*p* = 0.664). The participants in the emotional eating with moderate restraint class had the lowest mean EDSS score (3.8 [SD 1.0]) and a comparatively shorter mean disease duration (9.2 [SD 5.7] years). The high emotional eating and restraint class contained the largest proportion of women (85.7%), whereas the dysregulated overeating class contained the smallest proportion of women (51.6%). As these comparisons were unadjusted, they are presented descriptively and should not be interpreted as independent associations.

### 3.2. Coping Styles and Health Locus of Control

The adjusted associations of coping styles and health locus of control with eating-related latent-class membership are presented in Table 3. In the model, including CISS coping styles, task-oriented coping (*p* < 0.001), emotion-oriented coping (*p* < 0.001), and avoidance-oriented coping (*p* = 0.014), each showed a global association with class membership after adjustment for sex, age, disease duration, and EDSS.

Compared with the low eating-related difficulties class, the moderate mixed tendencies class was associated with higher task-oriented coping (relative risk ratio [RRR] = 1.96, 95% confidence interval [CI] 1.28–3.02; *p* = 0.002). The emotional eating with moderate restraint class was associated with lower task-oriented coping (RRR = 0.35, 95% CI 0.21–0.61; *p* < 0.001) and higher emotion-oriented (RRR = 2.82, 95% CI 1.77–4.47; *p* < 0.001) and avoidance-oriented coping (RRR = 1.96, 95% CI 1.12–3.45; *p* = 0.019). The dysregulated overeating class was associated with lower task-oriented coping (RRR = 0.53, 95% CI 0.29–0.96; *p* = 0.036) and higher avoidance-oriented coping (RRR = 2.21, 95% CI 1.18–4.14; *p* = 0.013). The high emotional eating and restraint class did not differ significantly from the reference class on any CISS dimension (all *p* ≥ 0.054).

All three MHLC dimensions also showed global associations with latent-class membership (all *p* < 0.001). Relative to the reference class, the emotional eating with moderate restraint class was associated with lower internal health control (RRR = 0.31, 95% CI 0.20–0.49; *p* < 0.001), stronger beliefs in the influence of powerful others (RRR = 3.17, 95% CI 1.94–5.19; *p* < 0.001), and stronger beliefs in the role of chance (RRR = 1.97, 95% CI 1.20–3.26; *p* = 0.008). Powerful-others health beliefs were also higher in the moderate mixed tendencies (RRR = 1.76, 95% CI 1.21–2.57; *p* = 0.003), high emotional eating and restraint (RRR = 1.75, 95% CI 1.10–2.81; *p* = 0.019), and dysregulated overeating (RRR = 2.05, 95% CI 1.16–3.62; *p* = 0.014) classes. No other class-specific contrasts for MHLC dimensions reached statistical significance.

### 3.3. Self-Management According to Eating-Related Latent Class

The adjusted Multiple Sclerosis Self-Management Scale-Revised (MSSM-R) total scores differed across the eating-related latent classes after adjustment for sex, age, disease duration, and EDSS (F(4, 373) = 3.81; *p* = 0.005; incremental R^2^ = 0.035; Table 4). Compared with the low eating-related difficulties class, the adjusted MSSM-R scores were higher in the emotional eating with moderate restraint class (99.6 vs. 94.2; B = 5.3, 95% confidence interval [CI] 1.8 to 8.9; *p* = 0.003) and the high emotional eating and restraint class (100.3 vs. 94.2; B = 6.0, 95% CI 1.8 to 10.3; *p* = 0.005).

The moderate mixed tendencies class did not differ significantly from the reference class (B = 2.2, 95% CI −1.1 to 5.4; *p* = 0.191). Similarly, the dysregulated overeating class did not differ significantly from the reference class (B = −0.8, 95% CI −5.7 to 4.1; *p* = 0.748).

The sensitivity analysis excluding MSSM-R Item 7, which assesses regular meal consumption, yielded a closely similar pattern (F(4, 373) = 3.70; *p* = 0.006; incremental R^2^ = 0.034). Relative to the low eating-related difficulties class, the emotional eating with moderate restraint class (B = 5.2, 95% CI 1.8 to 8.6; *p* = 0.003) and the high emotional eating and restraint class (B = 5.7, 95% CI 1.6 to 9.8; *p* = 0.006) again had higher adjusted self-management scores, whereas the moderate mixed tendencies and dysregulated overeating classes did not differ significantly from the reference class.

The classification-uncertainty sensitivity analysis produced the same substantive pattern (Table S1). After pseudo-class draws from the posterior membership probabilities, self-management remained higher in the emotional eating with moderate restraint class (B = 5.2, 95% CI 1.6 to 8.8; *p* = 0.005) and the high emotional eating and restraint class (B = 5.5, 95% CI 1.1 to 9.9; *p* = 0.013), whereas the remaining contrasts did not reach statistical significance. The corresponding findings were unchanged after exclusion of the regular-meals item.

## 4. Discussion

This study identified five distinct configurations of eating-related tendencies among adults with multiple sclerosis (MS): low eating-related difficulties, moderate mixed tendencies, emotional eating with moderate restraint, high emotional eating with restraint, and dysregulated overeating. These classes were not arranged on a simple continuum from low to high overall difficulty. Rather, they differed in the relative prominence of habitual overeating, emotional overeating, and dietary restraint. The retained solution, therefore, supports a person-centred interpretation of eating-related tendencies in MS and suggests that similar total levels of difficulty can conceal qualitatively different response configurations.

The identified heterogeneity is compatible with contemporary conceptualisations of emotional eating as a broad tendency rather than a unitary process. Emotional states can be associated with either increased or reduced food intake, and associations between stress, emotions, and eating are shaped by individual, situational, and measurement-related factors [5,6,20]. Systematic reviews likewise indicate that stress and negative affect are associated with disruptions in eating behaviour, although the direction and magnitude of these associations are variable across individuals and contexts [21,22]. Accordingly, the present profiles should not be interpreted as diagnostic categories or as direct indicators of eating-disorder pathology. They represent patterns of self-reported behavioural tendencies that may arise from different psychological and environmental processes.

The findings also fit with person-centred research showing that eating behaviours commonly aggregate into distinguishable configurations rather than operate as independent dimensions. Studies of children, young adults, parents, postpartum women, and treatment-seeking adults have reported profiles characterised by relatively low or moderate difficulties, food-approach tendencies, emotional eating, restraint, avoidance, or dysregulated eating [8,9,10,11,23]. These studies differ substantially in populations, instruments, and analytical specifications, so they do not imply the existence of a universal taxonomy. Nevertheless, they support the value of examining combinations of eating-related behaviours when the research question concerns heterogeneity among individuals. In the present study, the use of all 30 dichotomous MEH items allowed the latent classes to be defined by item-response configurations instead of pre-defined subscale totals, which is consistent with recommendations for model-based classification when indicators are categorical [7].

The emotional eating with moderate restraint class showed the clearest pattern of psychological correlates. Relative to the low-difficulties class, membership in this class was associated with lower task-oriented coping, higher emotion-oriented and avoidance-oriented coping, lower internal health locus of control, and stronger beliefs in the influence of powerful others and chance. This profile may indicate that emotional eating and dietary-control efforts coexist with an emotion-focused and externally oriented way of responding to illness-related demands. The interpretation must remain cautious: the cross-sectional data cannot establish whether these coping and control-belief patterns precede, accompany, or follow eating-related tendencies. However, the direction of the associations is broadly consistent with the MS literature in which perceived stress, emotion-focused coping, and certain avoidant responses have been linked to poorer adjustment, whereas problem-focused coping has more often been associated with more favourable psychosocial outcomes [24,25,26,27].

The health locus of control findings add a further layer to this interpretation. Health-control beliefs are not inherently adaptive or maladaptive; their implications may depend on illness demands, perceived controllability, coping options, and the treatment context. In MS, recent large-scale evidence has shown that health locus of control is associated with a range of clinical and psychosocial variables, while earlier work linked internal control and problem-oriented coping with better mental health and external control with more emotion-oriented coping [12,28]. The present associations, therefore, support the view that eating-related tendencies can be embedded in broader systems of illness appraisal and coping. They do not demonstrate that changing health-control beliefs would change eating behaviours, or vice versa.

The coexistence of emotional eating and dietary restraint in two profiles is also clinically relevant. Restraint should not be viewed as a uniform indicator of healthy self-control, nor should emotional eating be assumed to denote a single behavioural mechanism. Research on eating pathology and emotion regulation suggests that difficulties in identifying, accepting, or regulating affect are associated with a range of eating-related symptoms and strategies, including overeating, rigid control attempts, rumination, and avoidance [29,30]. In this context, the two restraint-containing profiles may reflect different configurations of efforts to regulate intake, affect, or health-related routines. The emotional eating with moderate restraint profile profile had a pronounced coping and locus-of-control pattern, whereas the high emotional eating and restraint profile did not differ significantly from the low-difficulties class on any class-specific CISS or MHLC contrast. Thus, elevated restraint had different psychological correlates depending on the broader eating-related configuration.

Because class membership explained only a small additional proportion of the variance in self-management (incremental R^2^ = 0.035), these differences should be interpreted cautiously. Higher self-management scores in both restraint-containing profiles require particularly careful interpretation. The MSSM-R assesses a broad set of illness-management activities, including knowledge, communication with healthcare professionals, symptom and treatment management, and responses to disease-related barriers; it is not a measure of dietary restraint [2,17]. The higher self-management scores observed in the two restraint-containing profiles may indicate that dietary restraint is embedded, for some patients, in a broader pattern of monitoring, planning, and engagement with illness-management routines. However, this should not be interpreted as evidence that restrictive eating is adaptive or that higher self-management protects against emotional eating. Rather, the finding suggests that self-management engagement and eating-related control may overlap behaviourally while differing in clinical meaning. The persistence of the difference after excluding the regular-meals item and after accounting for uncertainty in class assignment reduces, but does not eliminate, concern that the result arose solely from direct content overlap. Previous MS research has similarly emphasised that self-management is related to psychological and coping processes, while intervention studies suggest potential benefits of self-management support for quality of life and selected psychological outcomes [13,31].

The dysregulated overeating profile was characterised by high habitual and emotional overeating with comparatively lower dietary restraint. It was associated with lower task-oriented coping, higher avoidance-oriented coping, and stronger powerful-others health beliefs, but not with a difference in self-management relative to the low-difficulties class. This pattern differed from both restraint-containing profiles and may represent a configuration in which eating-related difficulties are more closely aligned with reduced behavioural regulation than with deliberate efforts to control food intake. Similar high-disinhibition or dysregulated profiles have been reported in treatment-seeking samples with compulsive eating or obesity, where they were also associated with more adverse psychological and eating-related characteristics [11]. Nonetheless, the present profiles cannot be equated with binge-eating disorder or another clinical diagnosis because this study did not assess objective binge episodes, loss of control, body mass index, dietary intake, or eating-disorder diagnoses. Its interpretation should, therefore, remain descriptive.

The moderate mixed tendencies class showed higher task-oriented coping together with stronger powerful-others health beliefs than the low-difficulties class. This finding cautions against treating beliefs in the role of healthcare professionals as a purely passive or unfavourable orientation. In MS, long-term treatment and rehabilitation often require continuing engagement with neurologists, rehabilitation professionals, and other sources of specialist support. It is plausible that, in some individuals, stronger powerful-others beliefs coexist with active collaboration in managing the condition. This explanation is necessarily tentative, but it is compatible with the broader view that the function of a health-control belief is contingent on the clinical setting and on the coping behaviours with which it is combined [12,28].

The findings should be interpreted as hypothesis-generating rather than as evidence for immediate clinical classification or intervention decisions. Eating-related questions may nevertheless be useful as part of a broader discussion of self-management, coping, and emotional responses to MS, especially when patients describe difficulties with avoidance, emotional regulation, or perceived personal control. The present profiles should not be used as diagnostic labels and do not identify people with eating disorders. Rather, they suggest that similar reports of eating-related difficulty may occur in different psychological contexts. For example, emotional eating accompanied by restraint may warrant attention to both dietary-control efforts and affective coping, whereas a dysregulated-overeating pattern may justify further assessment of routines, avoidance, and perceived barriers to behavioural self-regulation. Any clinical response should be individualised and integrated with neurological, nutritional, and psychological care. This cautious interpretation is consistent with evidence that psychosocial factors, coping, self-management support, and quality of life are closely interrelated in MS [27,31,32]. Because the present study did not assess dietary intake, body weight or BMI, body composition, fatigue, relapse activity, inflammatory markers, clinical diagnoses of depression, or eating-disorder diagnoses, the clinical significance of these profiles requires prospective validation before they can be used to guide patient care.

Several strengths of this study should be noted. The analysis used all 30 MEH items, enabling classification to reflect item-level response configurations rather than only subscale scores. The five-class solution was selected after consideration of fit indices, classification quality, class sizes, replication of the best log-likelihood value, and substantive interpretability. Associations with coping and health locus of control were estimated using one-step latent class regression models, thereby avoiding the assumption that class membership is measured without error. The self-management analyses were adjusted for sex, age, disease duration, and neurological disability and were robust to both the removal of the regular-meals item and the pseudo-class sensitivity analyses that incorporated classification uncertainty. These features align with recommended safeguards for interpreting latent class solutions and their external correlates [7].

This study also has limitations. First, the cross-sectional design prevents conclusions about temporal ordering and causality. Eating-related tendencies, coping, health-control beliefs, and self-management may be reciprocally associated over time. In addition, the parent dataset did not include detailed measures of dietary intake, body weight or BMI, body composition, relapse activity, inflammatory markers, or fatigue; therefore, the present analyses cannot determine whether the identified profiles are associated with nutritional status, disease activity, or other clinical outcomes. Clinical diagnoses of depression were also not assessed, and mood-related variables were not analysed as clinical outcomes in this secondary analysis. Second, the participants were recruited from two specialist clinical settings in Poland, which may limit generalisability to community samples, patients treated in other healthcare systems, and people with different levels of disability or healthcare access. In addition, individuals with documented psychotic disorder or clinically apparent cognitive impairment were not invited when these conditions were judged likely to compromise informed consent or reliable questionnaire completion. The findings should, therefore, not be generalised to these groups without further study. Third, the MEH is a Polish self-report questionnaire designed to characterise eating-related tendencies rather than diagnose eating disorders. Although it was appropriate for the present secondary analysis and for item-level Bernoulli latent class modelling, its international validation is more limited than that of widely used instruments, such as the Dutch Eating Behaviour Questionnaire and the Three-Factor Eating Questionnaire. The dichotomous response format facilitated modelling of endorsed item-level tendencies, but may have reduced sensitivity to differences in severity. In addition, the restrictive-dieting subscale showed lower internal consistency than the other MEH dimensions [4]. Several external correlate scales also showed modest internal consistency, particularly internal health locus of control, chance health locus of control, and avoidance-oriented coping, which may have attenuated associations and should be considered when interpreting non-significant findings. Fourth, self-report methods may be susceptible to recall, response-style, and shared-method biases. Finally, the latent classes are model-based summaries of response patterns in this sample, not fixed patient types. Although classification uncertainty was addressed in sensitivity analyses for the self-management comparisons, descriptive class summaries based on modal assignment may still be affected by imperfect classification. Replication in independent MS cohorts and prospective validation against dietary, nutritional, psychological, and clinical outcomes are required.

## 5. Conclusions

In conclusion, adults with MS showed heterogeneous patterns of habitual overeating, emotional overeating, and dietary restraint. The emotional eating with moderate restraint class was characterised by lower task-oriented coping, higher emotion-oriented and avoidance-oriented coping, lower internal health control, and stronger external control beliefs. Both restraint-containing classes had higher self-management scores, whereas the dysregulated overeating class showed lower task-oriented coping and higher avoidance-oriented coping without a self-management difference. For people with MS, this study does not provide dietary or psychological treatment recommendations, but it shows that eating-related difficulties should not be assumed to represent a single severity continuum or a uniform clinical problem. For the research field, this study provides an empirically derived five-profile structure and shows that these profiles differ in coping, health-control beliefs, and self-management. This allows future studies to be designed differently: rather than analysing eating-related tendencies only as separate subscale scores, prospective research can test the stability of these profiles and examine whether they predict dietary intake, nutritional status, mood, fatigue, quality of life, disability progression, or other clinical outcomes. Until such validation is available, the profiles should be regarded as descriptive and exploratory.

## Figures and Tables

**Table 1:** Fit indices for Bernoulli latent class models of eating-related tendencies (N = 382).

Classes	Log-Likelihood	Parameters	AIC	BIC	SABIC	Entropy	Smallest Class, n (%)
1	−6384.544	30	12,829.088	12,947.450	12,852.265	—	382.0 (100.0)
2	−5822.763	61	11,767.526	12,008.196	11,814.653	0.890	167.5 (43.9)
3	−5355.780	92	10,895.559	11,258.538	10,966.637	0.943	44.6 (11.7)
4	−5181.326	123	10,608.652	11,093.939	10,703.680	0.962	31.8 (8.3)
5	−5076.005	154	10,460.009	11,067.604	10,578.988	0.920	31.0 (8.1)
6	−5006.366	185	10,382.732	11,112.635	10,525.661	0.933	17.1 (4.5)
7	−4935.693	216	10,303.387	11,155.597	10,470.266	0.934	16.0 (4.2)

Note. AIC, the Akaike information criterion; BIC, the Bayesian information criterion; SABIC, the sample-size-adjusted BIC. Entropy is reported for solutions with two or more classes. The five-class solution was retained because it had the lowest BIC, high classification quality, an adequate smallest estimated class (approximately 31 participants), and substantively interpretable response patterns. The seven-class solution did not improve the BIC or substantive interpretability. The values are based on a Bernoulli latent class analysis of the 30 dichotomous MEH items.

**Table 2:** Demographic and clinical characteristics according to modal membership in the five eating-related latent classes.

Characteristic	Overall (N = 382)	Low Difficulties (n = 93)	Moderate Mixed Tendencies (n = 115)	Emotional Eating + Moderate Restraint (n = 94)	High Emotional Eating + Restraint (n = 49)	Dysregulated Overeating (n = 31)	*p* Value
Female sex, n (%)	256 (67.0)	60 (64.5)	76 (66.1)	62 (66.0)	42 (85.7)	16 (51.6)	0.022
Age, years, mean (SD)	46.4 (11.9)	47.8 (12.9)	46.2 (12.1)	46.2 (11.4)	45.0 (11.7)	45.3 (9.7)	0.664
Disease duration, years, mean (SD)	11.5 (9.1)	13.8 (11.8)	12.8 (9.4)	9.2 (5.7)	9.5 (6.8)	10.5 (8.0)	0.002
EDSS, mean (SD)	4.4 (1.7)	4.8 (1.8)	4.7 (1.8)	3.8 (1.0)	4.1 (1.8)	4.2 (1.7)	<0.001
MS clinical course, n (%)							0.006 *
Relapsing-remitting MS	158 (41.4)	28 (30.1)	44 (38.3)	44 (46.8)	27 (55.1)	15 (48.4)	
Secondary progressive MS	70 (18.3)	20 (21.5)	19 (16.5)	21 (22.3)	6 (12.2)	4 (12.9)	
Primary progressive MS	88 (23.0)	26 (28.0)	20 (17.4)	25 (26.6)	9 (18.4)	8 (25.8)	
Progressive-relapsing MS	33 (8.6)	10 (10.8)	14 (12.2)	3 (3.2)	5 (10.2)	1 (3.2)	
Unknown course	33 (8.6)	9 (9.7)	18 (15.7)	1 (1.1)	2 (4.1)	3 (9.7)	

Note. The values are the mean (SD) unless otherwise indicated. The *p* values are unadjusted and were obtained from one-way analysis of variance for age, disease duration, and EDSS; Pearson chi-square test for sex; and a Monte Carlo exact test for MS clinical course. * the Monte Carlo *p* value. Class membership was assigned using the highest posterior probability; consequently, the class sizes differ slightly from the model-estimated prevalences reported in Table 1. EDSS = Expanded Disability Status Scale; MS = multiple sclerosis.

**Table 3:** Adjusted associations of coping styles and health locus of control with membership in eating-related latent classes.

Predictor	*p* for Global Association	Moderate Mixed Tendencies RRR (95% CI); *p*	Emotional Eating + Moderate Restraint RRR (95% CI); *p*	High Emotional Eating + Restraint RRR (95% CI); *p*	Dysregulated Overeating RRR (95% CI); *p*
Panel A. Coping styles (CISS)					
Task-oriented coping	<0.001	1.96 (1.28–3.02) 0.002	0.35 (0.21–0.61) <0.001	1.28 (0.77–2.15) 0.342	0.53 (0.29–0.96) 0.036
Emotion-oriented coping	<0.001	1.00 (0.66–1.52) 0.989	2.82 (1.77–4.47) <0.001	1.55 (0.93–2.58) 0.092	1.50 (0.89–2.53) 0.131
Avoidance-oriented coping	0.014	1.03 (0.68–1.56) 0.883	1.96 (1.12–3.45) 0.019	1.70 (0.99–2.92) 0.054	2.21 (1.18–4.14) 0.013
Panel B. Health locus of control (MHLC)					
Internal control	<0.001	0.99 (0.70–1.40) 0.958	0.31 (0.20–0.49) <0.001	0.99 (0.62–1.57) 0.952	0.61 (0.36–1.03) 0.062
Powerful others	<0.001	1.76 (1.21–2.57) 0.003	3.17 (1.94–5.19) <0.001	1.75 (1.10–2.81) 0.019	2.05 (1.16–3.62) 0.014
Chance	<0.001	0.70 (0.49–1.00) 0.053	1.97 (1.20–3.26) 0.008	1.32 (0.80–2.15) 0.275	1.53 (0.85–2.78) 0.160

Note. The RRRs and 95% CIs were estimated in two separate one-step latent class regression models: one including the three CISS coping styles and one including the three MHLC dimensions. Each model was adjusted for sex, age, disease duration, and EDSS. The low eating-related difficulties class was the reference outcome category. The focal psychological predictors were standardised; therefore, the RRRs represent the relative risk of class membership associated with a 1-SD higher score. *p* values beneath the RRRs refer to class-specific contrasts with the reference class. The *p* for global association is the 4-df Wald test for the association of a predictor with latent-class membership across all non-reference classes. RRR = the relative risk ratio; CI = the confidence interval; CISS = Coping Inventory for Stressful Situations; MHLC = Multidimensional Health Locus of Control; EDSS = Expanded Disability Status Scale.

**Table 4:** Adjusted multiple sclerosis self-management scores according to membership in eating-related latent classes.

Eating-Related Latent Class	n	Adjusted Mean (SE)	Difference Versus Low Difficulties, B (95% CI); *p*
Panel A. MSSM-R total score (24 items): F(4, 373) = 3.81; *p* = 0.005; incremental R^2^ = 0.035			
Low eating-related difficulties	93	94.2 (1.2)	Reference
Moderate mixed tendencies	115	96.4 (1.1)	2.2 (−1.1 to 5.4); 0.191
Emotional eating + moderate restraint	94	99.6 (1.3)	5.3 (1.8 to 8.9); 0.003
High emotional eating + restraint	49	100.3 (1.7)	6.0 (1.8 to 10.3); 0.005
Dysregulated overeating	31	93.4 (2.1)	−0.8 (−5.7 to 4.1); 0.748
Panel B. Sensitivity analysis: MSSM-R score excluding Item 7 (regular meals): F(4, 373) = 3.70; *p* = 0.006; incremental R^2^ = 0.034			
Low eating-related difficulties	93	90.2 (1.2)	Reference
Moderate mixed tendencies	115	92.4 (1.1)	2.2 (−0.9 to 5.4); 0.164
Emotional eating + moderate restraint	94	95.4 (1.2)	5.2 (1.8 to 8.6); 0.003
High emotional eating + restraint	49	95.9 (1.7)	5.7 (1.6 to 9.8); 0.006
Dysregulated overeating	31	89.5 (2.1)	−0.7 (−5.4 to 4.0); 0.783

Note. The adjusted means are estimated marginal means from the linear regression models controlling for sex, age, disease duration, and EDSS; covariates were held at their sample means. The differences and 95% CIs compare each class with the low eating-related difficulties reference class in the same model. The incremental R^2^ denotes the increase in explained variance after adding the 4-df class term to the covariate-only model. Class membership was assigned using the highest posterior probability. MSSM-R = Multiple Sclerosis Self-Management Scale-Revised; EDSS = Expanded Disability Status Scale. The sensitivity score excluded Item 7 (“I make sure to eat regular meals”), which overlaps conceptually with eating-related behaviour. The classification-uncertainty sensitivity analyses are reported in Supplementary Table S1.

## Data Availability

The data presented in this study are available on reasonable request from the corresponding author.

## Supplementary Materials

The following supporting information can be downloaded at: https://www.mdpi.com/article/10.3390/nu18182984/s1. Table S1: Classification-uncertainty sensitivity analyses of multiple sclerosis self-management according to eating-related latent class.
