# Supplementary material for: Eating-Related Profiles in Multiple Sclerosis: Associations with Coping, Health Locus of Control, and Self-Management
Source: Nutrients. 2026 Sep 11;18(18):2984. doi: 10.3390/nu18182984 (PMC13610605; doi:10.3390/nu18182984)
Supplement: Supplementary file 1 [file nutrients-18-02984-s001.zip › nutrients-4533269-supplementary.pdf]

Table S1. Classification-uncertainty sensitivity analyses of multiple sclerosis self-management according to eating-related latent class.

Eating-related latent class	Modal n	Adjusted mean (SE)	Difference versus low difficulties, B (95% CI); P
**Panel A. MSSM-R total score (24 items)**			
**Low eating-related difficulties**	93	94.4 (1.3)	Reference
**Moderate mixed tendencies**	115	96.3 (1.1)	1.8 (-1.7 to 5.3); .305
**Emotional eating + moderate restraint**	94	99.7 (1.3)	5.2 (1.6 to 8.8); .005
**High emotional eating + restraint**	49	100.0 (1.8)	5.5 (1.1 to 9.9); .013
**Dysregulated overeating**	31	93.4 (2.1)	-1.1 (-6.0 to 3.9); .671
**Panel B. Sensitivity score excluding MSSM-R Item 7 (regular meals)**			
**Low eating-related difficulties**	93	90.4 (1.3)	Reference
**Moderate mixed tendencies**	115	92.3 (1.1)	1.9 (-1.5 to 5.2); .283
**Emotional eating + moderate restraint**	94	95.5 (1.2)	5.0 (1.5 to 8.5); .005
**High emotional eating + restraint**	49	95.6 (1.7)	5.2 (0.9 to 9.4); .017
**Dysregulated overeating**	31	89.5 (2.1)	-0.9 (-5.7 to 3.8); .698

Note. Adjusted means and contrasts are derived from a pseudo-class sensitivity analysis that sampled class membership from individual posterior probability vectors across 2,000 draws. Each draw was analysed with a linear regression adjusted for sex, age, disease duration, and EDSS; estimates and variance components were pooled using Rubin's rules. Modal class counts are displayed only to describe the observed class distribution. MSSM-R = Multiple Sclerosis Self-Management Scale-Revised; EDSS = Expanded Disability Status Scale.
